# Hierarchical sparse spatiotemporal graph neural network for brain graph classification

**DOI:** 10.1016/j.isci.2026.116173

**Published:** 2026-06-04

**Authors:** Jiaqi Cui, Yuxin Li, Xiran Qu, Yupei Zhang

**Affiliations:** 1 School of Computer Science, Northwestern Polytechnical University, Xi’an, China; 2 Laboratory of Big Data Storage and Management, Ministry of Industry and Information Technology, Xi’an, China; 3 Institute of Flexible Electronics, Northwestern Polytechnical University, Xi’an, China

**Keywords:** neuroscience, computational bioinformatics

## Abstract

Brain graph classification from resting-state fMRI (rs-fMRI) can support the identification of neurological conditions and inform personalized analysis. Here, we present a hierarchical sparse spatiotemporal graph neural network (STGNN)—GLNSTGNN—to address sparse feature selection in spatiotemporal brain graph classification. We evaluated GLNSTGNN on two rs-fMRI datasets comprising 1,956 participants with 200 regions of interest (ROIs) and 12 subnetworks after standardized preprocessing. GLNSTGNN applies GroupLassoNet-based hierarchical sparsity to select informative features, while combining spatial graph convolution on a fixed functional connectivity adjacency with temporal convolution on time-varying BOLD signals to capture spatial dependencies and temporal dynamics. Across multiple baselines, GLNSTGNN showed improved discriminative performance and consistent ROI selection, supporting interpretable subnetwork-level patterns. These results suggest that integrating hierarchical sparsity with spatiotemporal graph learning can provide a practical framework for robust and interpretable brain graph classification.

## Highlights

• GLNSTGNN integrates hierarchical sparsity for spatiotemporal feature selection
• L1/L2,1 hierarchical sparsity supports interpretable ROI-to-subnetwork patterns
• GLNSTGNN adaptively learns discriminative substructures without hard thresholding
• On ABIDE and ADHD rs-fMRI, GLNSTGNN identifies biologically meaningful ROIs

## Graphical abstract

**Figure:**
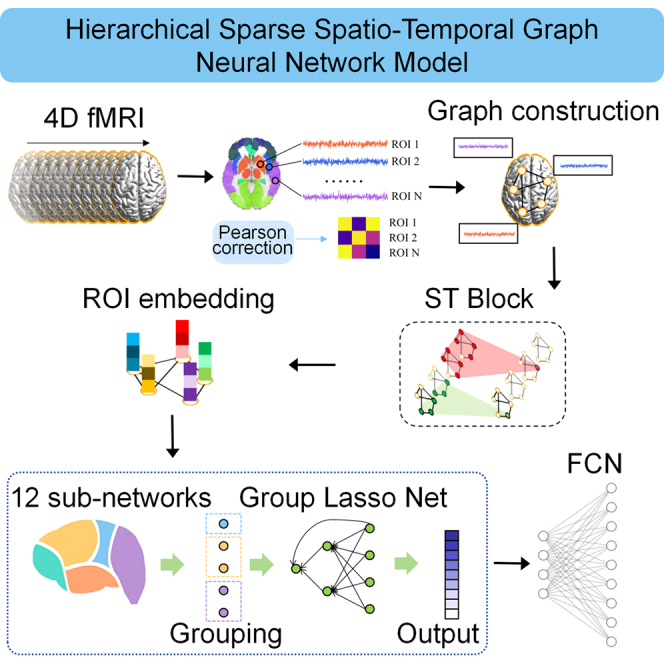

## Introduction

Brain graph analysis maps the structural or functional connections of the human brain into a graphical representation, modeling neural connectivity through graph-theoretic formulations.^1^^,^^2^ Brain graph classification has become an important topic at the intersection of computer science and neuroscience, serving as a key foundation for personalized medicine and early diagnosis.^3^ By extracting structural or functional features, evaluating inter-group brain differences,^4^ and identifying discriminative brain regions,^5^ these studies support early disease detection and precision healthcare. Current research mainly focuses on extracting discriminative functional or structural features from brain graphs.^6^^,^^7^^,^^8^ Although machine learning and deep learning methods have achieved promising results,^9^ challenges such as high-dimensionality, data noise, and nonlinear connectivity remain. Recent advances have demonstrated that combining machine learning and deep learning techniques can significantly enhance feature representation and pattern recognition across various domains. For example, TransIFC^10^ proposed an invariant cue-aware feature concentration learning approach for fine-grained bird image classification, effectively enhancing discriminative localization in visual recognition. Similarly, DSR-Net^11^ introduced a distinct selective rollback mechanism for road crack detection based on detection transformers, achieving high robustness in complex outdoor environments. In addition, UGENet^12^ developed a self-attention-based discriminative embedding framework for unconstrained gaze estimation in human-computer interactions, demonstrating the adaptability of deep learning architectures to multimodal perceptual tasks. These studies collectively illustrate that deep neural networks can extract invariant, fine-grained, and domain-adaptive features, providing valuable insights for our work into brain graph modeling. However, in the context of brain network analysis, challenges such as high-dimensionality, inter-subject variability, and difficulty in capturing hierarchical spatiotemporal dependencies still remain.

Given the complex topology of brain networks, researchers have increasingly adopted graph neural networks (GNNs) to learn high-level representations from neuroimaging data.^13^ Early works, such as ChebNet,^14^ applied spectral graph convolution for brain graph analysis, which was later optimized by the graph convolutional networks (GCNs) to improve computational efficiency.^15^ Subsequent studies built upon these foundations; Liu et al.^16^ and Qin et al.^17^ used Pearson’s correlation matrices as node features and employed Chebyshev graph convolutions for neurological disorder prediction. Cao et al.^18^ further extended GCNs to deeper architectures (16 layers) for enhanced hierarchical feature extraction, while Ma et al.^19^ combined GCN-derived graph features with phenotypic information for improved classification. More recently, several brain-specific GNN architectures have been proposed to enhance interpretability and biological plausibility. For example, BrainGNN^20^ introduces region of interest (ROI)-aware convolution layers and selection pooling to improve interpretability, while SAGN^21^ employs sparse adaptive gating to capture dual-view (strongly vs. weakly coupled) brain networks with graph regularization. FBNetGen^22^ further learns task-oriented brain networks through an end-to-end functional network generation framework. However, these models primarily focus on spatial graph adaptation or ROI selection, lacking explicit hierarchical sparsity across layers. In contrast, our proposed GLNSTGNN integrates spatiotemporal convolution with hierarchical sparse constraints, achieving both spatiotemporal feature selection and structured interpretability.

To better capture dynamic brain activity, spatiotemporal GNNs (STGNNs) have been introduced.^23^ Xing et al.^24^ constructed dynamic functional networks, using a sliding window approach, feeding ROI volume data from T1-MRI into LSTM modules. Building on this, Kim et al.^25^ integrated GCN-based spatial encoding with LSTM-based temporal modeling, while Yao et al.^26^ combined graph convolution within each resting-state fMRI (rs-fMRI) segment and convolutional neural network (CNN)-based temporal extraction between segments.^27^^,^^28^ These advances demonstrate a transition from RNN-based to CNN-based temporal modeling frameworks. However, current STGNNs still struggle to model smoothly evolving graph topologies,^29^ highlighting the need for robust hierarchical representations capable of capturing both spatial dependencies and temporal continuity.

Changes in brain graph structures often arise from noise or irrelevant factors, while disease-related subnetworks remain sparse and stable. To improve interpretability, sparse learning has been extensively explored.^30^ The classical Lasso^31^ enforces feature-level sparsity, while group Lasso^32^ extends this to predefined feature groups. Li et al.^33^ applied adaptive group sparsity for genetic-based cancer detection. More recent frameworks such as LassoNet^34^ embed *ℓ*_1_ regularization directly into neural networks for joint feature selection and learning. Zhang et al.^35^ utilized group sparsity to enhance inter-group separability and intra-group consistency in brain functional networks, and Gong et al.^36^ developed an online sparse decomposition approach to extract shared and individual-specific connectivity patterns. However, most existing sparse learning methods focus solely on independent feature selection, neglecting inter-feature and inter-layer dependencies crucial for brain network modeling. Recent studies in representation learning and perception modeling have shown that explicitly capturing inter-feature dependencies can significantly enhance discriminative capability and model interpretability. For example, MMATrans^37^ introduced a muscle movement-aware transformer framework for facial expression recognition, where correlated muscle activations were modeled to improve feature expressiveness. LDCNet^38^ employed local dense connectivity to enhance inter-layer feature propagation for image recognition, demonstrating that feature interactions across scales contribute to robustness and efficiency. Similarly, EHPE^39^ involved a skeleton cue-based Gaussian coordinate-encoding mechanism for human pose estimation, effectively capturing structural correlations between joints. Inspired by these findings, our proposed hierarchical sparse strategy differs fundamentally from adaptive sparsity methods like SAGN^21^ or FBNetGen,^22^ as it explicitly enforces structured sparsity both within and across groups over spatiotemporal graph features, thereby achieving interpretable and stable subnetwork discovery. By integrating both intra-group (*ℓ*_1_) and inter-group (*ℓ*_2,1_) regularization, GLNSTGNN jointly models feature relevance and dependency across spatial and temporal dimensions, enhancing both interpretability and generalization stability.

To address these challenges, we propose a group-wise and layer-wise normalized STGNNN (GLNSTGNN). GLNSTGNN introduces hierarchical sparse regularization that combines intra-group (*ℓ*_1_) and inter-group (*ℓ*_2,1_) penalties, allowing simultaneous spatiotemporal feature selection and network-level interpretability. Unlike traditional hard-threshold feature selection, GLNSTGNN adaptively learns discriminative substructures and preserves essential spatiotemporal dependencies. Through real-world evaluations on the ABIDE and ADHD rs-fMRI datasets, GLNSTGNN not only achieves strong predictive performance but also identifies biologically meaningful ROIs, providing valuable insights for precision neuroscience and disease diagnosis.

The input $X\in R^{n\times v\times T}$ represents the BOLD time series of ROIs, where *n* denotes the sample size, *v* is the number of ROIs, and *T* is the length of the time series. Each sample $x\in R^{v\times T}$ represents the ROI-by-time BOLD signal matrix, where each row is an ROI time series over *T* time points. The target output is the prediction label $Y\in R^{n\times 1}$.

## Results

### Hierarchical sparse STGNN

**Figure 1:**
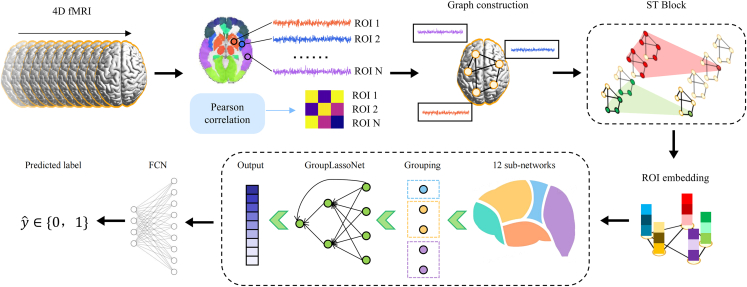
Framework of the hierarchical sparse STGNN: GLNSTGNN

The design of the hierarchical sparse STGNN—GLNSTGNN—is shown in Figure 1. First, the correlation coefficients are used to calculate the correlations between various ROIs in *x*, constructing a static graph. Subsequently, spatial convolution and temporal convolution operations are applied to the extracted spatiotemporal features to obtain *x*_*e*_ = STGNN (*x*), where $x_{e}\in R^{v\times d}$. Spatial convolution is primarily employed to capture the spatial dependency relationships between different brain regions, while temporal convolution aims to capture time-varying patterns within the BOLD time series over time.

Next, the node embedding representation *x*_*e*_ is fed into the hierarchical sparse neural network. The input features *x*_*e*_ are divided into groups *G*_*roup*_ = [*g*_1_, *g*_2_, …, *g*_*m*_] and passed through the neural network architecture *W*. This network employs a residual neural network framework with skip connections from the input to the output, where the parameters of the skip layers are denoted as *θ*.

To achieve feature selection, the algorithm imposes two regularization constraints on the weights of the skip connections: first, the *L*_1_ norm is used to enforce intra-group sparsity; second, the *L*_2_ norm is applied to enhance inter-group sparsity. Through these regularization constraints, the model can not only select the most relevant features but also achieve sparsity both within and across groups, thereby improving the interpretability and generalization ability of the model. All parameters are first initialized and updated via ordinary gradient descent, and then the parameters (*θ*, *W*^(1)^) are further optimized according to the proximal gradient descent principle until the convergence criteria are met.

Finally, after being processed by the hierarchical sparse neural network, the extracted features are fed into a fully connected network for final classification. The fully connected layer generates predictive outputs for classification by integrating information from the preceding layers. The sparse graph generated by this network can highlight key brain network connections relevant to the prediction task, providing unique and in-depth interpretations for neuroscience research.

### Performance

#### Subnetwork grouping supports development of classification model

**Figure 2:**
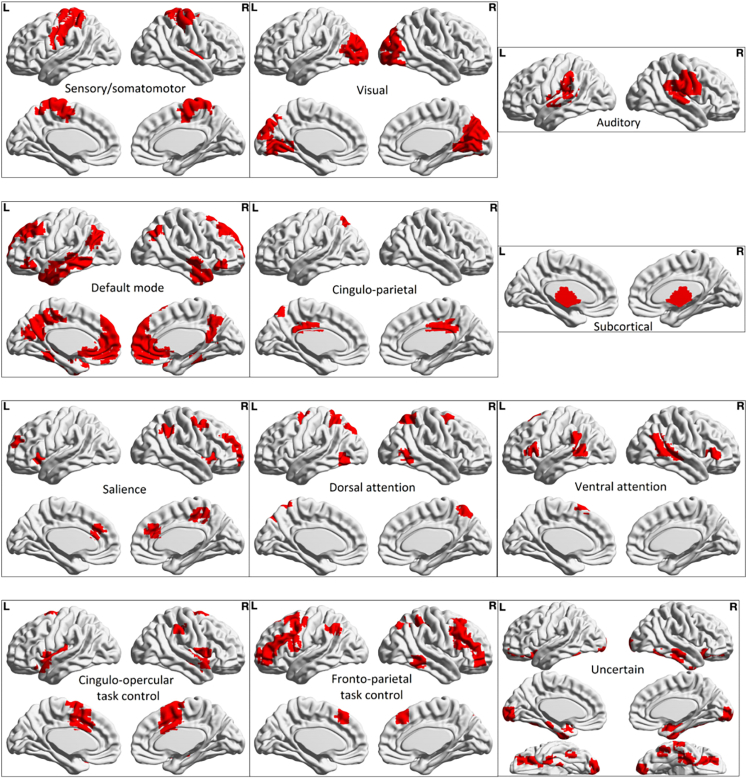
ROIs related to the 12 subnetworks used

A deep understanding of discriminative connective subnetworks is crucial for clinical interpretability. In the field of neuroscience, the correct identification and grouping of different brain region subnetworks can provide an important biological basis for the early diagnosis of diseases and the optimization of treatment strategies. To reasonably group 200 nodes, we identified twelve different subnetworks, which cover various brain functional modules from perception to cognitive control, as shown in Figure 2. Specifically, the identified subnetworks include the following categories: sensory/somatomotor hand (containing 15 ROIs), sensory/somatomotor mouth (containing 3 ROIs), cingulo-opercular task control (containing 12 ROIs), auditory (containing 9 ROIs), default mode (containing 41 ROIs), cingulo-parietal (containing 3 ROIs), visual (containing 20 ROIs), fronto-parietal task control (containing 24 ROIs), salience (containing 10 ROIs), subcortical (containing 25 ROIs), ventral attention (containing 8 ROIs), dorsal attention (containing 13 ROIs), and uncertain (containing 17 ROIs; this network includes brain regions that do not belong to any defined network). The division of these subnetworks is based on the functional connectivity characteristics of ROIs. Through the systematic classification and analysis of the topological structure of the brain network, it provides a clearer perspective for understanding the inter-relationships of the brain functional modules.

In the process of constructing the dataset, these brain region networks initially proposed by Power et al.^40^ have been widely applied in neuroimaging research. To apply these subnetworks to a more refined brain atlas, this study adopted the Craddock 200 cortical network template (CC200) atlas, a functional connectivity analysis-based atlas that covers functional connectivity data of 200 brain regions. The specific method was to match each ROI in the CC200 atlas with the corresponding ROI in the Power264 atlas one-to-one and classify each ROI into the corresponding network based on the network allocation rules of the Power264 atlas.^40^ The matching criteria used the minimum Euclidean distance algorithm, which can ensure that the ROIs in the CC200 and Power264 atlases have the smallest spatial position deviation, thus guaranteeing the accuracy of cross-atlas analysis.

To further ensure the accuracy of network allocation, all network allocation processes have undergone manual verification. This process not only validates the spatial overlap degree between ROIs in the atlases but also ensures the high anatomical and functional consistency of ROIs in the two atlases through visual inspection and numerical comparison. Such a rigorous verification process provides reliable basic data for subsequent experiments, making the research results more credible and clinically interpretable.

In addition, the study also paid special attention to the MNI coordinates of each region in the CC200 atlas (i.e., the coordinate positions of each brain region in the standard brain space), as well as the corresponding ROI names, volumes (in *mm*^3^), and assigned subnetwork types. This information provides a solid foundation for subsequent neural network analysis, functional connectivity pattern recognition, and their clinical applications. Detailed data, such as CC200 atlas regions, MNI coordinates, ROI names, and volume information, are provided in Table 1.

**Table 1:** Description of ROI-related information for part of the CC200 atlas^a^

ROI number	MNI coordinates (*x*, *y*, *z*)	ROI name	Volume (mm^3^)	Subnetwork
1	(−40.7, −21.3, 54.3)	Postcentral_L	235	sensorimotor/body movement (hand)
2	(−8.8, −38.0, 69.3)	Paracentral_Lobule_L	215	sensorimotor/body movement (hand)
3	(30.4, −33.7, 63.4)	Postcentral_R	240	sensorimotor/body movement (hand)
4	(43.3, −19.9, 53.7)	Precentral_R	266	sensorimotor/body movement (hand)
5	(12.3, −44.8, 67.7)	Postcentral_R	254	sensorimotor/body movement (hand)
⋮	⋮	⋮	⋮	⋮
36	(−56.0, −10.9, 5.6)	Temporal_Sup_L	230	auditory
37	(54.2, −11.9, 39.3)	Postcentral_R	224	auditory
38	(44.5, −24.8, 16.1)	Rolandic_Oper_R	222	auditory
39	(−41.9, −31.5, 15.2)	Temporal_Sup_L	211	auditory
40	(−49.7, −60.8, 23.2)	Angular_L	269	default mode
⋮	⋮	⋮	⋮	⋮
196	(28.3, 43.8, −13.5)	Frontal_Mid_Orb_R	67	uncertain
197	(−21.1, −91.8, −12.1)	Occipital_Inf_L	236	uncertain
198	(9.0, −86.9, −12.3)	Lingual_R	249	uncertain
199	(30.8, −1.4, −36.2)	Fusiform_R	157	uncertain
200	(−15.0, −30.8, −18.3)	ParaHippocampal_L	173	uncertain

a ⋮ indicates omitted rows/entries for brevity; the CC200 atlas contains 200 ROIs in total, and only a subset is shown here.

#### Comparison of different models in brain image classification

Table 2 shows the performance evaluation results of various classification methods on the ABIDE and ADHD datasets, covering indicators such as AUC, ACC, SEN, PPV, F1, and SPE. GLNSTGNN is the method proposed in this study, and CNN,^41^ FCNet,^42^ LDS,^43^ GTS,^44^ FBNetGen, BrainNetCNN,^45^ BrainNetTF,^46^ and RostGNN^47^ are the comparative methods. All AUC values are reported with 95% confidence intervals estimated using DeLong’s method. The following research findings can be drawn.

**Table 2:** The classification performance of all methods under multiple indicators^a^

Method	ABIDE						ADHD					
	AUC (95% CI)	ACC	SEN	PPV	F1	SPE	AUC (95% CI)	ACC	SEN	PPV	F1	SPE
CNN	55.6 [51.9–58.8]	53.9	62.4	54.3	58.1	45.1	55.6 [52.0–58.9]	53.0	46.1	40.1	42.9	57.3
FCNet	59.3 [55.0–63.0]	56.6	61.0	57.1	59.0	52.0	56.7 [52.2–60.2]	55.2	56.8	43.4	49.2	54.2
LDS	67.0 [62.6–71.0]	66.2	68.0	66.6	67.3	64.3	63.2 [59.0–67.8]	62.9	69.5	51.1	58.9	58.8
GTS	68.8 [63.3–72.9]	65.9	75.3	64.2	69.3	56.0	64.1 [59.9–67.9]	63.5	67.5	51.7	58.6	61.0
FBNetGen	70.5 [65.8–74.7]	68.0	71.2	67.8	69.5	64.6	65.3 [61.0–69.2]	65.6	67.2	54.0	59.9	64.6
BrainNetCNN	72.1 [68.0–75.7]	67.4	72.6	66.6	69.5	61.9	66.8 [63.0–70.1]	67.3	69.3	55.8	61.8	66.0
BrainNetTF	67.8 [66.4–69.5]	66.5	67.7	67.2	67.6	63.4	62.9 [62.0–65.2]	62.2	65.3	52.1	58.8	59.2
RostGNN	73.7 [69.8–77.2]	70.4	**75.7**	69.3	**72.4**	64.9	65.9 [62.4–69.3]	69.6	**70.0**	58.6	**63.8**	69.4
GLNSTGNN	**75.9 [73.6-78.3]**	**77.4**	62.3	**79.6**	69.6	**88.3**	**74.7 [71.9-76.5]**	**71.6**	54.4	**66.6**	58.5	**82.4**

a Bold entries in Table 2 indicate the best performance among all methods for each metric on the corresponding dataset.

On the ABIDE dataset, GLNSTGNN performed optimally in key metrics such as AUC (75.9%), ACC (77.4%), PPV (79.6%), and SPE (88.3%), significantly outperforming other methods. For example, its AUC was 2.2 percentage points higher than that of the second-best method, RostGNN (73.7%). Although it was slightly inferior to RostGNN (75.7%) in SEN (62.3%), the high PPV of GLNSTGNN indicates its prominent advantage in accurate classification. In contrast, traditional methods like CNN and FCNet have AUC values of only 55.6% and 59.3%, with ACC at 53.9% and 56.6%, respectively, and their performance lagged significantly those of GLNSTGNN and RostGNN. More complex comparative methods such as FBNetGen, BrainNetCNN, and BrainNetTF, despite improvements in some metrics, had overall performance inferior to that of GLNSTGNN.

On the ADHD dataset, GLNSTGNN again demonstrated excellent performance, with AUC, ACC, PPV, and SPE reaching 74.7%, 71.6%, 66.6%, and 82.4%, respectively, all being significantly higher than those of other methods. Compared with RostGNN, the AUC of GLNSTGNN increased by 8.8 percentage points (from 65.9% to 74.7%), and the ACC increased by 2 percentage points (from 69.6% to 71.6%). Although its SEN (54.4%) was slightly lower than that of RostGNN (70.0%), its advantages in PPV and SPE further verify its accurate classification ability. Traditional methods such as CNN and FCNet still performed poorly, while the improvements of BrainNetCNN and FBNetGen on the ADHD dataset were limited and failed to surpass GLNSTGNN and RostGNN.

Longitudinal comparison showed that GLNSTGNN exhibited strong consistency on both datasets and remained leading in key metrics such as AUC, ACC, PPV, and SPE. This indicates the effectiveness of its method design and generalization ability across different tasks. Pairwise DeLong tests indicated that the improvements over RostGNN (*p* = 0.021), BrainNetCNN (*p* = 0.009), and other baselines were statistically significant (*p* < 0.006). These results confirmed that GLNSTGNN representation yields a significant enhancement in discriminative ability over existing models. In contrast, RostGNN excelled in SEN and F1, especially on the ADHD dataset, but its overall performance was still inferior to that of GLNSTGNN. Traditional methods like CNN and FCNet performed poorly on both datasets, while complex methods such as BrainNetCNN and FBNetGen performed relatively better on ABIDE but had poor adaptability on the ADHD dataset. In addition, the overall classification performance of the ADHD dataset was generally lower than that of the ABIDE dataset, indicating that the ADHD task is more complex.

In summary, GLNSTGNN demonstrated significantly better classification performance than other methods on the ABIDE and ADHD datasets, particularly excelling in key metrics such as AUC, ACC, PPV, and SPE, which fully verify the robustness and efficiency of its method. These results further prove the superiority of GLNSTGNN.

**Figure 3:**
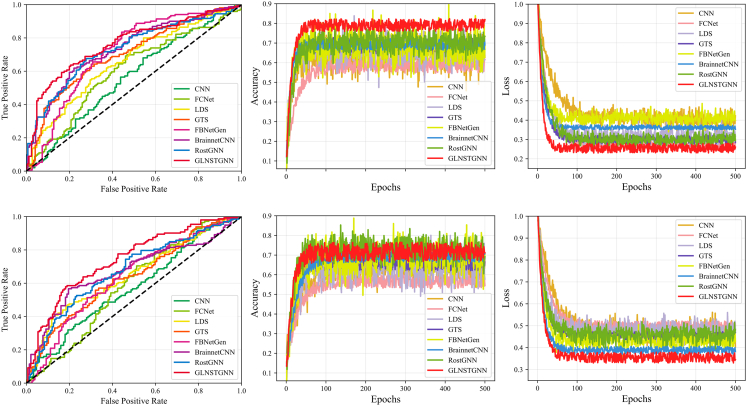
Comparison and analysis of the performance of multiple models

As can be seen from the ROC curves in the first column of Figure 3, the AUC value of GLNSTGNN was significantly better than that of the other methods on both the ABIDE and ADHD datasets. The curve is closer to the ideal upper-left corner, indicating its extremely strong classification ability. From the perspective of the accuracy change curve, GLNSTGNN has a fast convergence speed, achieving high accuracy in the early training rounds, and leading among all methods in the final accuracy, demonstrating strong generalization ability and adaptability to complex data. In the loss change curve, the loss of GLNSTGNN decreases the fastest and is smooth and stable, reflecting an efficient optimization process. Overall, GLNSTGNN is significantly better than other methods in both classification performance and optimization efficiency, especially excelling in key indicators such as AUC and accuracy, which prove its strong capability and design innovation in brain network classification tasks.

#### Gaussian noise experiment

**Figure 4:**
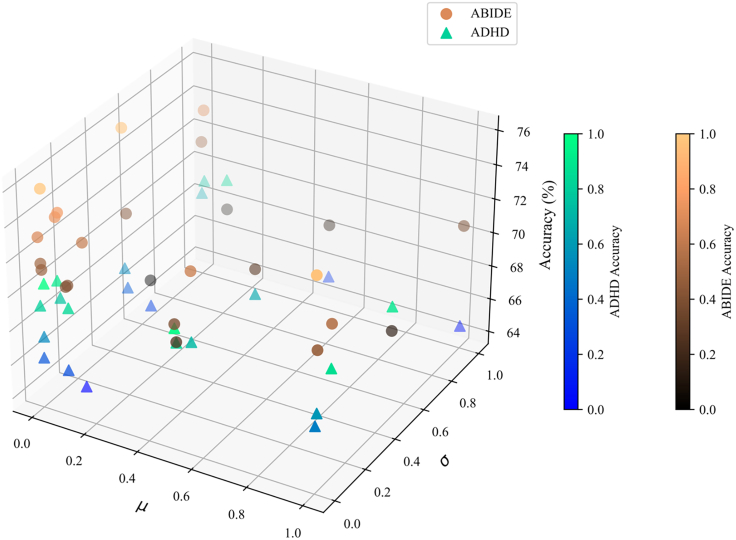
Gaussian noise experiment

It can be seen from Figure 4 that different Gaussian noise parameters *μ* and *σ* have a significant impact on the classification accuracy of the ABIDE and ADHD datasets. With the increase in noise intensity, the classification accuracy generally showed a downward trend, but high accuracy could still be maintained under specific parameter combinations, indicating that the model is robust to noise within a certain range. The accuracy on the ABIDE dataset was higher than that on ADHD under various noise conditions, showing its stronger adaptability to noise and more stable classification performance. This experiment showed that the proposed method has strong anti-noise ability and exhibits high robustness and adaptability both on the ABIDE dataset with clear structure and the more challenging ADHD dataset. This robustness provides reliable technical support for processing brain network data that may be disturbed by noise in practice and further verifies the effectiveness and practical value of the proposed method in complex environments.

#### Ablation experiment

**Figure 5:**
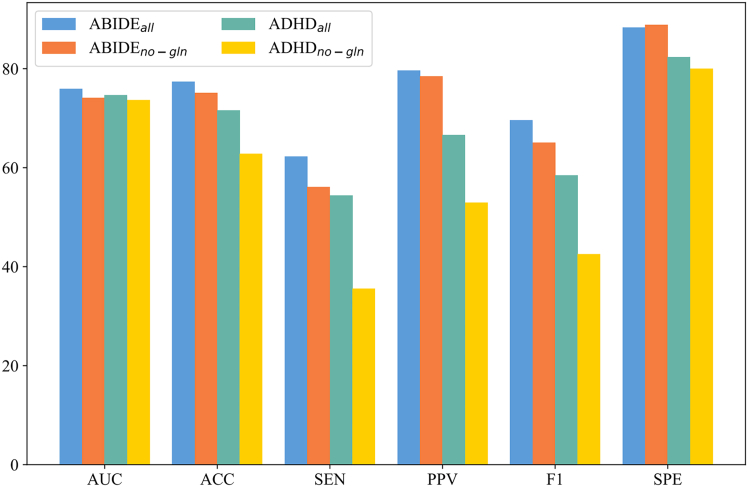
Ablation experiment results of GLNSTGNN

As can be seen from the ablation experiment results in Figure 5, for the GLNSTGNN method on both the ABIDE and ADHD datasets, when all modules are included (*all*), the performance is significantly better than the case where the GroupLassoNet module is removed (*non* − *gln*). Specifically, on the ABIDE dataset, when the GroupLassoNet module is included (ABIDE_*all*_), the model performs optimally in indicators such as AUC, ACC, PPV, F1, and SPE, with particularly significant improvements in ACC and SPE. This indicates that the introduction of the GroupLassoNet module can significantly enhance the model’s ability to distinguish between positive and negative categories, while improving its ability to filter non-target categories. A similar trend could be observed on the ADHD dataset. The model with the GroupLassoNet module (ADHD_*all*_) performed better than the model without this module (ADHD_*no*−*gln*_) in terms of indicators such as AUC, ACC, PPV, and SPE. Although it was slightly inferior in term of the SEN indicator, this may be because GroupLassoNet pays more attention to the balance of overall accuracy, emphasizing the accurate identification of positive categories while taking into account the ability to filter negative categories. In summary, the ablation experiment verifies the effectiveness and necessity of the GroupLassoNet module. The introduction of this module not only improves the model’s performance in multiple key indicators but also enhances the model’s robustness and generalization ability when processing complex brain network data.

#### Sparse structure visualization

In addition to improving the classification performance, another core goal of the proposed model design was to enhance the interpretability of results. To this end, an experiment was designed, as discussed in this section, to identify the brain regions that play a key role in distinguishing autism spectrum disorder (ASD) and attention deficit hyperactivity disorder (ADHD) from healthy samples. As mentioned earlier, the GroupLassoNet module has the ability to identify and retain important nodes during training, while effectively discarding redundant or irrelevant nodes. This module receives 200 node embeddings in the graph, where each node represents an independent brain region. By applying hierarchical sparse feature selection to these graphs, the model can remove some nodes and generate a sequence of sparse output graphs with fewer nodes. In the experiment, 5-fold cross-validation was used to train the GLNSTGNN model, and then the output of the pooling layer of the test dataset was stored and analyzed. By counting the frequency of nodes in the output of the pooling layer, the 5 nodes with the highest frequency were finally selected as important regions for diagnosing brain diseases. This process demonstrates the model’s advantages in feature selection and result interpretation as well as helps reveal important brain regions related to neuropathology.

**Figure 6:**
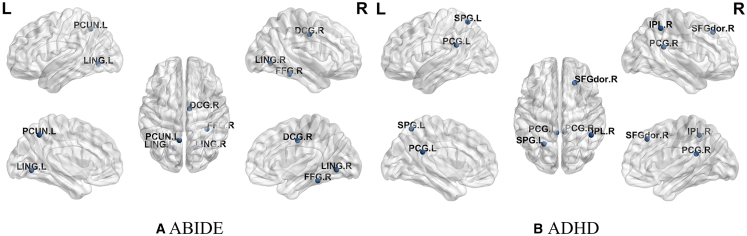
5 key regions for diagnosing brain diseases through sparse substructure identification The experiment was conducted separately on the ABIDE and ADHD datasets. The five key ROIs selected by the GLNSTGNN were rendered on a standard brain surface using the BrainNet Viewer toolbox for qualitative interpretation. (A) shows the ROIs identified for ABIDE, and (B) shows the ROIs identified for ADHD-200.

This experiment was conducted on the ABIDE and ADHD-200 datasets, and the 5 identified key brain regions were visualized using the BrainNet Viewer toolbox, as shown in Figure 6. The experimental results showed that in autism diagnosis, the lingual cortex is the most influential brain region, and this finding is consistent with existing research results.^48^ In addition, other identified important regions include the cingulate cortex, fusiform cortex, and precuneus cortex. These regions have also been considered in previous studies to be closely related to the pathogenesis of autism.^49^ Similarly, in the diagnosis of ADHD, the parietal cortex, cingulate cortex, and frontal cortex were identified as key regions, which is highly consistent with the research results of Bush et al.,^50^ supporting the biological plausibility of the identified regions and the interpretability of the model outputs.

**Figure 7:**
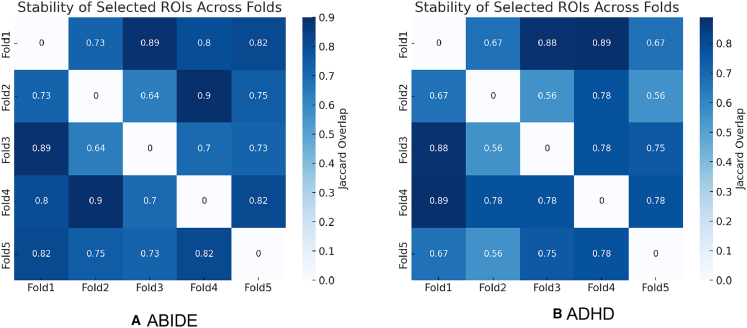
Stability of selected ROIs across 5-fold cross-validation for (A) ABIDE and (B) ADHD datasets

To further evaluate the interpretability robustness of the proposed GLNSTGNN, we assessed the consistency of the selected ROIs across the 5-fold cross-validation experiments. As shown in Figure 7, the pairwise Jaccard heatmaps visualize the degree of overlap among folds. For the ABIDE dataset, the average Jaccard stability index was 0.738 ± 0.104, indicating that most discriminative ROIs were consistently selected across folds. For the ADHD dataset, the mean Jaccard index was 0.729 ± 0.111, suggesting slightly greater variability in the identified regions, possibly due to the higher heterogeneity and site-specific variability inherent in ADHD cohorts. Overall, these results demonstrated that GLNSTGNN achieves stable and reproducible feature selection across folds, supporting the stability and reproducibility of ROI selection across cross-validation folds. GLNSTGNN differs from widely used post-hoc explainers such as GNNExplainer,^51^ which typically provides instance-wise explanations by learning a sparse subgraph/feature mask that maximizes the prediction for a specific subject, and from gradient-based visualization methods such as Grad-CAM,^52^ which highlight influential nodes/regions via gradients or activation maps for a given prediction. While post-hoc explainers can offer complementary subject-level insights, they may be sensitive to optimization/initialization and can produce variable explanations across runs.

## Discussion

### The impact of sparse parameters

**Figure 8:**
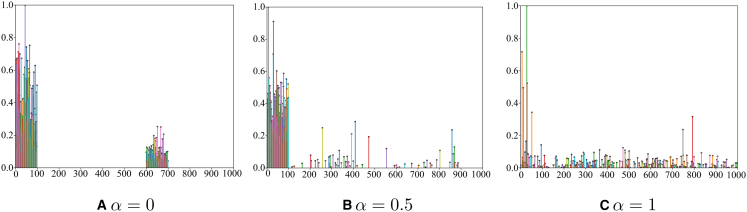
The influence of parameter *α* on self-expressive sparse representation (A) (α = 0). Feature-weight distribution when only inter-group sparsity is enforced. Most within-group features remain dense, while only a small number of groups retain non-zero responses, reflecting group-level selection without intra-group pruning. (B) (α = 0.5). Feature-weight distribution under combined inter-group and intra-group sparsity. Non-zero weights concentrate on a small subset of features within the most relevant group, while irrelevant groups/features are largely suppressed, indicating balanced hierarchical sparsification. (C) (α = 1). Feature-weight distribution under strong intra-group sparsity.

To explore the impact of the trade-off parameter *α* in Equation 4 on sparsity, we conducted systematic parameter experiments based on the experimental configuration in Figure 8. In the experiment, a group sparse self-expression method was adopted, where similar samples of the same class were grouped together. The goal was to study the effect of feature selection under different parameter settings by fitting any image in the test set. Specifically, a random image of class “0” was selected as the target sample and fitted. To intuitively show the impact of *α* on sparsity, the control algorithm terminated training after selecting approximately 100–200 features. The reason for choosing this specific range is that in the actual experimental data, the number of feature samples in each class is 100; ideally the algorithm should be able to accurately identify these 100 features to better fit the test samples.

During the experiment, by controlling the value of parameter *α*, the sparse distribution of features within and between groups could be clearly observed. The experimental results are shown in Figure 8, which presents the feature sparsity distribution under the condition of *α* = {0, 0.5, 1}, where the height of each line represents the magnitude of the feature weight. Based on Equation 4, when *α* = 0, as shown in Figure 8A, sparsification occurred only between groups, that is, the image features of class “0” and class “6” were retained, while almost all features within groups were preserved. In this case, the algorithm tends to retain complete category features. Relatively, when *α* = 1, each group showed a certain degree of sparsity, among which the weight of class “0” features was more prominent. Although the output results were still concentrated on the target group, many target features were missed. This indicates that the introduction of group structure constraints can significantly improve the effect of feature extraction. When *α* ∈ (0, 1), the algorithm could sparsify both intra-group and inter-group features, as shown in Figure 8B. Under this condition, most of the non-zero weights were concentrated in the feature range (0 − 100) of class “0,” but some features of other classes were still retained. However, category features like class “9” were completely removed.

It can be concluded from the experimental results that different *α* values have a significant impact on the focus of sparsification. When the *α* value is small, the algorithm tends to retain intra-group features and highlight inter-group sparsity; when the *α* value is large, both intra-group and inter-group sparsity are significantly enhanced, and in this case, the features of non-target categories are significantly suppressed.

**Figure 9:**
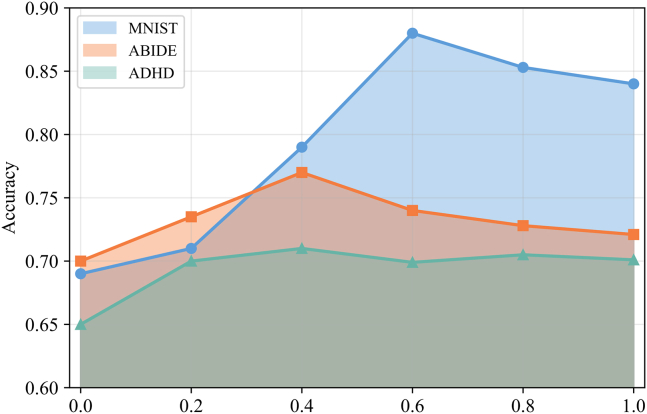
Classification accuracy corresponding to different trade-off parameters *α*

In this simulation experiment, the focus was on extracting target features. However, to ensure the discriminative ability of the extracted features, the classification accuracy after feature extraction by GroupLassoNet was further tested. Figure 9 depicts the trend of test accuracy under different *α* parameter values on three datasets: MNIST, ABIDE, and ADHD. It can be clearly seen from the figure that when *α* ∈ (0, 1), the test accuracy was higher than that when *α* = 0 or *α* = 1. This result indicates that appropriately balancing the intra-group and inter-group sparsity can significantly improve the classification performance. Specifically, when the *α* value is small, although the inter-group sparsity performs strongly, it may retain too many redundant features within the group, resulting in the failure to fully improve the classification performance; when the *α* value is large, excessive sparsification between and within groups may lead to the loss of effective features, thereby reducing the classification performance. Therefore, a moderate *α* value can make full use of the inherent structure of the data in feature selection, which not only reduces the interference of irrelevant features on the model but also retains sufficient discriminative information.

In summary, by adjusting the trade-off parameter *α*, GroupLassoNet can achieve a balance between intra-group and inter-group sparsity, thereby demonstrating excellent performance in the classification tasks. These results demonstrate the practical utility of the sparse trade-off strategy within the evaluated experimental settings and may inform its use in applications involving high-dimensional sparse data.

### Sensitivity analysis on window size parameter

**Figure 10:**
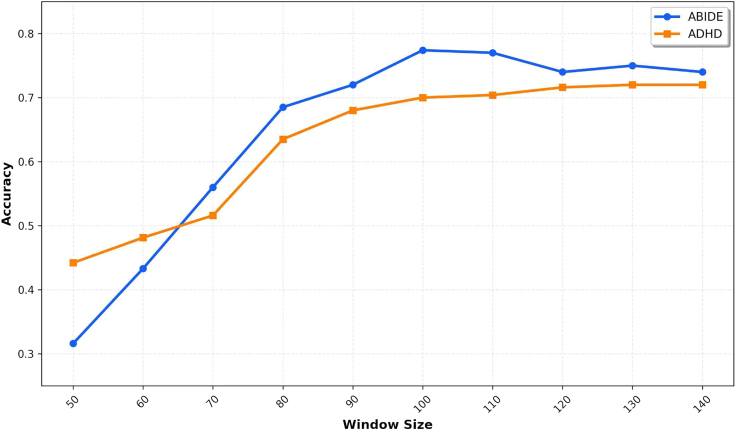
Sensitivity analysis on window size

To further understand the effect of window size on the model’s performance, we conducted a sensitivity analysis across different window sizes. As shown in Figure 10, we evaluated the classification accuracy on the ABIDE and ADHD datasets by varying the window size. The accuracy of the model steadily increased as the window size grew, with a noticeable plateau at a window size of approximately 120. This trend suggests that larger window sizes enable the model to capture richer temporal patterns in node signals on a fixed graph, leading to better classification performance. Interestingly, accuracy on the ABIDE dataset exhibited a sharper increase than the ADHD dataset, which may indicate inherent differences in data structure or task complexity between these datasets. Slower improvement on the ADHD dataset may also suggest that the model benefits less from larger windows, potentially due to the noise or variability in the data, which could obscure temporal variations in the BOLD signals. Additionally, while the model’s accuracy continued to rise with the increasing window size, diminishing returns were observed beyond a certain threshold. This indicates an optimal window size around 120, beyond which performance gains become marginal. These findings highlight the importance of tuning window size to optimize classification performance and computational efficiency. This sensitivity analysis provides a better understanding of the trade-offs involved in choosing window and threshold parameters and guides further model optimization for different datasets.

### Evaluation of cross-site generalization

**Figure 11:**
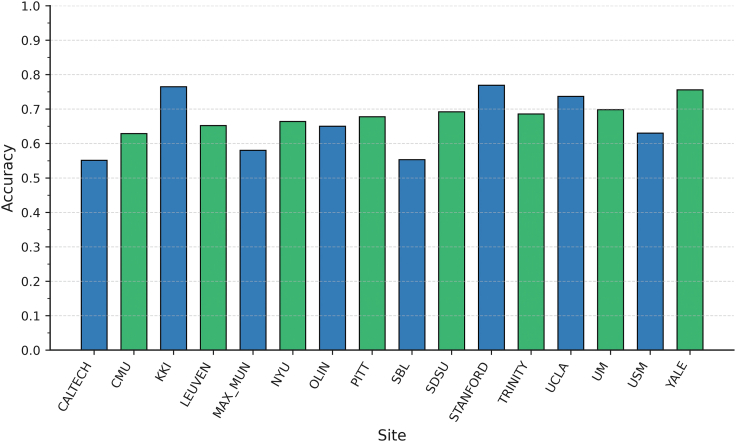
Leave-one-site-out 5-fold cross-validation results using GLNSTGNN

To evaluate the generalization performance of the GLNSTGNN model across multiple sites, we conducted a leave-one-site-out (LOSO) cross-validation procedure, where each site was sequentially excluded as the test set, while the remaining sites were used for training. This setup enables the model’s robustness and adaptability to be assessed under realistic cross-site variation. As illustrated in Figure 11, the model achieved relatively consistent accuracy across sites, demonstrating strong generalization capability. Slight performance differences among the sites can be explained by factors such as participants’ age distribution, sample size, and data quality. For instance, sites with larger or younger average age datasets (e.g., UCLA and SDSU) tend to yield higher accuracies, whereas sites with smaller or more heterogeneous samples (e.g., CALTECH and SBL) exhibit reduced performance. These observations suggest that inter-site variability, while inevitable in multi-center neuroimaging datasets, can influence predictive outcomes. Nevertheless, GLNSTGNN showed consistent performance across sites, demonstrating resilience to site-related variability in the LOSO evaluation and maintaining comparable performance across diverse data sources.

### Limitations of the study

Several limitations should be noted. First, ABIDE and ADHD-200 differ in their acquisition protocols and scan durations. Accordingly, we estimated the functional connectivity (FC), using the full, available rs-fMRI time series within each dataset. While this avoids non-equivalent temporal coverage introduced by aggressive truncation, FC estimation may still be influenced by dataset-specific sequence length and protocol differences, which should be considered when interpreting cross-dataset comparisons. Future work will further examine length-matched and harmonized settings across cohorts to isolate acquisition-related effects. Second, our validation was conducted on a limited number of public datasets; evaluations on larger multi-center cohorts and additional disorders are important to better assess robustness and generalization. Third, the current model relies on a static FC graph and does not explicitly model time-varying connectivity. Extending GLNSTGNN to dynamic or windowed connectivity and incorporating temporal attention mechanisms may further improve performance in scenarios where transient topology changes are critical.

## Resource availability

### Lead contact

Requests for further information and resources should be directed to and will be fulfilled by the lead contact, Yupei Zhang (ypzhaang@nwpu.edu.cn).

### Materials availability

This study did not generate new unique reagents.

### Data and code availability

• This paper analyzes existing, publicly available data. These accession numbers for the datasets are listed in the key resources table.
• Code is available at https://github.com/JiaqiCUI-npu/GLNSTGNNCode.
• Any additional information required to reanalyze the data reported in this paper is available from the lead contact upon request.

## Acknowledgments

This study was supported by the 10.13039/501100001809National Natural Science Foundation of China (nos. 62272392 and 62433016), the 10.13039/501100015401Key Research and Development Program of Shaanxi Province (no. 2023-YBGY-405), and Key Educational Reform Project of 10.13039/501100002663Northwestern Polytechnical University (no. 2024JGZ21).

## Author contributions

J.C. and Y.L. designed the study under the supervision of Y.Z.; J.C. and Y.L. implemented the models, performed the experiments, and wrote the manuscript; X.Q. assisted with data preprocessing and participated in helpful discussions during the early stage of this work; Y.Z. administered the project and provided funding support.

## Declaration of interests

The authors declare no competing interests.

## STAR★Methods

### Key resources table

**Table:** 

REAGENT or RESOURCE	SOURCE	IDENTIFIER
**Deposited data**		

Code	This paper	https://github.com/JiaqiCUI-npu/GLNSTGNNCode.git
ABIDE Dataset	This paper	https://fcon_1000.projects.nitrc.org/indi/abide
ADHD Dataset	This paper	https://fcon_1000.projects.nitrc.org/indi/adhd200

**Software and algorithms**		

Python 3.8.0	Python	https://www.python.org
TensorFlow 1.9.0	TensorFlow	https://tensorflow.org
PyTorch (v1.12.1+cu113)	PyTorch	https://pytorch.org
CUDA Toolkit (v11.3, runtime)	NVIDIA	https://developer.nvidia.com/cuda-toolkit
Conda (Anaconda/Miniconda)	Anaconda, Inc.	https://docs.conda.io

### Experimental model and study participant details

#### Study participants

No new participants, samples, or experimental models were recruited or generated for this study. All analyses were conducted using de-identified, publicly available rs-fMRI datasets from the Preprocessed Connectomes Project (PCP), including ABIDE and ADHD-200. Participant demographics and inclusion/exclusion criteria are defined by the original studies and the PCP preprocessing pipelines, and are summarized in the corresponding dataset documentation.

#### Ethics statement

Because this study used publicly available, de-identified data, no additional institutional review board approval or informed consent was required for the analyses performed in this work.

### Method details

#### Spatio-temporal convolution module

The Spatio-Temporal Graph Convolutional Network (ST-GCN) effectively processes structured time-series data by integrating graph convolutions in the spatial dimension to capture local spatial dependencies and convolutional operations along the temporal dimension to mine temporal dependencies between adjacent time points. The spatio-temporal graph convolution module combines graph and temporal convolutions to extract useful spatio-temporal features from raw data,^28^ as illustrated in Figure S1. Specifically, edges in the spatial dimension represent Pearson correlations between ROI time series computed over the full rs-fMRI scan yielding a static FC adjacency, while edges in the temporal dimension connect signal values of the same graph node across two consecutive time points.

Spectral graph theory extends traditional convolution operations from grid-structured data to graph-structured data. In this study, brain networks naturally exhibit graph structures, where the features of each node can be regarded as signals on the graph. Therefore, to fully utilize the topological properties of brain networks, graph convolution operations based on spectral graph theory are to directly process signals, so as to explore the signal correlations of brain networks in the spatial dimension. By transforming graphs into algebraic forms, spectral graph methods can effectively analyze the topological properties of graphs, such as the connectivity of graph structures.

In the spatial domain, it is assumed that the node features *x* and the adjacency matrix $A\in R^{v\times v}$ together form the graph representation of the brain. At this point, the input to the spatio-temporal graph convolutional network is the spatio-temporal signal $X\in R^{C\times v\times T}$, where *C* denotes the number of channels in the network. The convolution formula for the spatial graph is as follows:

(Equation 1) $$Z^{l}=\left(D^{-\frac{1}{2}}\left(A+I\right)D^{-\frac{1}{2}}\right)F^{l-1}W_{\text{SG}}$$

In this context, $A=D^{-\frac{1}{2}}\left(A+I\right)D^{-\frac{1}{2}}$ denotes the normalized adjacency matrix, **D** is the degree matrix of nodes, and **I** is the identity matrix, which adds self-loops to each node. **Z**^*l*^ represents the convolutional features at the *l*-th layer, with the initial condition **Z**^(0)^ = *X*. $W_{\text{SG}}\in R^{C\times M}$ is the convolution kernel for the spatial graph, and *M* denotes the number of output channels at the *l*-th layer.

After the graph convolution operation captures the neighborhood information of each node, standard temporal convolution layers are further stacked to merge information from adjacent temporal segments of the node signals and update the node signals, as illustrated on the right side of Figure 2. Specifically, in the temporal dimension, since the temporal graph exhibits a regular grid structure, the time-series features $Z_{i}\in R^{M\times T}$ of node *v*_*i*_ undergo standard 1D convolution:

(Equation 2) $$Z_{i}^{l}=Z_{i}⊕W_{\text{TG}}$$

Where $Z_{i}^{l}$ is the feature output by node *v*_*i*_ at layer *l*, $W_{\text{TG}}\in R^{M\times \Gamma }$ is the temporal convolution kernel, and Γ is the size of the 1D convolution kernel.

The spatio-temporal convolution module can effectively capture the spatio-temporal features of data. By stacking multiple spatio-temporal convolution modules, the network can extract broader spatio-temporal correlations in the ROI (node) signals on a fixed FC graph. Finally, a fully connected layer is added to ensure that the outputs of each component have the same dimension and shape. The final fully connected layer uses ReLU as the activation function to enhance the network’s nonlinear modeling capability. With this structure, the spatio-temporal graph convolution network can capture deep-level dependency relationships in the spatio-temporal domain and achieve effective performance improvements in brain network analysis and other spatio-temporal sequence modeling tasks.

#### Hierarchical sparse network

To construct an associative network with hierarchical sparsity characteristics, we propose a deep sparse learning framework named GroupLassoNet, which integrates prior hierarchical structure information into the learning process. In many real-world scenarios, data exhibit complex hierarchical organizations that are often known or can be inferred from domain knowledge. Conceptually, correlations between different hierarchical levels are weak, whereas correlations within the same level are strong. By leveraging the principles of sparse learning, group-sparse regularization (combining *ℓ*_1_ and *ℓ*_2_ penalties) is applied to each layer of the neural network, enabling the model to automatically select and learn features most relevant to the target task. This design enforces hierarchical sparsity across layers, allowing GroupLassoNet to reveal potential associative relationships and improve both interpretability and generalization.

The ordinary residual neural network aims to learn the mapping function shown in Equation 3, where the parameter *θ* belongs to the skip layer, and *W* is the weight of any form of neural network. The mapping *g*_*W*_(*x*) is typically implemented by a fully connected feedforward network. Drawing on this idea, GroupLassoNet integrates an objective function for controlling sparsity and introduces sparsity constraints through residual connections of skip layers. Figure S2 illustrates the model design framework of GroupLassoNet in feature sparse learning.

(Equation 3) $$F=\left\{f\equiv f_{\theta ,W}:x\mapsto \theta^{T}x+g_{W}\left(x\right)\right\}$$

The objective function of GroupLassoNet is defined as shown in Equation 4. Here, *L*(⋅) represents an arbitrary loss function, and this paper selects the mean squared error (MSE); the parameter *λ* is the regularization coefficient, also known as the sparsity control parameter; *α* is used to balance the sparsity within groups and between groups, referred to as the trade-off parameter; and *M* serves as the Hierarchy Multiplier, adjusting the constraint strength of the skip layer on the feature input.

(Equation 4) $$\begin{array}{c} \underset{\theta ,W}{min}L\left(\theta ,W\right)+\lambda \cdot \left(\alpha \|\theta {\|}_{1}+\left(1-\alpha \right)\underset{g\in G}{\sum }\|\theta^{\left(g\right)}{\|}_{2}\right)\\ \;\text{s.t.}\;\|W_{j}^{\left(1\right)}{\|}_{\infty }\leq M|\theta_{j}|,\;j=1,\ldots ,d \end{array}$$

This method realizes the sparsity control of the skip layer through the first line of Equation 4, while the second line further restricts the weights of feature inputs by constraining the skip layer parameter *θ*. If *θ*_*j*_ = 0, then *W*_*j*_ = 0. Therefore, the sparsity regulation of the skip layer directly determines the sparsity of feature selection, which is in line with the core concept of LassoNet.

The objective function of this algorithm still belongs to the combination of “differentiable + non-differentiable” structure, so the idea of proximal gradient descent can be borrowed to solve it. However, due to the additional constraint condition, the derivation process is different. Based on the basic principle of proximal gradient descent and the internal additivity of 4, the core of the algorithm lies in deriving the global optimal solution of the optimization problem in 5 and processing it on a grouped basis.

(Equation 5) $$\begin{array}{cc} \min_{b\in R^{k},W\in R^{k\times k}} & \frac{1}{2}\left(\|r-b{\|}_{2}^{2}+\frac{1}{2}\left(\|U-W{\|}_{F}^{2}\right)\right)+\alpha \lambda \|b{\|}_{1}+\left(1-\alpha \right)\lambda \|b{\|}_{2}\\ s.t. & \|W_{i}{\|}_{\infty }\leq M|b_{i}|,i=1,2,\ldots ,k \end{array}$$

Where $r\in R^{k}$ and $U\in R^{k\times K}$ are known constant vectors or matrices, and *k* denotes the number of features in a certain group *g*_*i*_ of the grouping *G*. *K* is the number of neurons in the first hidden layer corresponding to the features within the group. Assume that the global optimal solution to the above optimization problem is (*b*∗, *W*∗).

Let *w* = *M* ⋅|*b*∗|, and the following is the proof of the assertion:

(Equation 6) $$\text{Claim}:W^{*}=\text{sign}\left(U\right)\cdot \min\left(w,|U|\right)$$

According to the definition, by eliminating the terms independent of *W* in 5, *W*∗ is the solution to the following optimization problem:

(Equation 7) $$\begin{array}{cc} \min_{W} & \frac{1}{2}\|U-W{\|}_{F}^{2}\\ s.t. & \|W_{i}{\|}_{\infty }\leq M|b_{i}^{*}|=w_{i},i\in k \end{array}$$

Next, for the dual variable $s\in R_{+}^{k}$, the Lagrangian function shown in 8 should be optimized:

(Equation 8) $$W^{*}=\mathrm{arg min}\left(\frac{1}{2}\|U-W{\|}_{2}^{2}+\overset{K}{\underset{j=1}{\sum }}s_{j}|W_{j}|\right)$$

Compute the subgradient of 8, and then combined with the KKT conditions, the desired *W*∗ needs to satisfy:

(Equation 9) $$\begin{array}{cc} W_{j}^{*}-U_{j}+s_{j}R_{j}^{*} & =0,\;\text{where}\;R_{j}^{*}=\partial \left(\left|W_{j}\right|\right)\\ s_{j}\left(|W_{j}^{*}|-w_{j}\right) & =0\\ s_{j} & \geq 0,\;|W_{j}^{*}|\leq w_{j} \end{array}$$

The above equation can be discussed in two cases:

1) When *s*_*j*_ = 0, *W*∗ = *U*, which holds ∗∗if and only if∗∗ *w* ≥|*W*∗| = |*U*|.
2) When *s*_*j*_ > 0, according to the KKT conditions (9), $|W_{j}^{*}|=w_{j}$. Since $R_{j}^{*}=sign\left(W_{j}^{*}\right)=sign\left(U_{j}\right)$ , it follows that *W*∗ = |*W*∗|⋅ *sign*(*W*∗) = *w* ⋅ *sign*(*U*). Combining with the principle of proximal gradual descent, we have *W*∗ = *S*_*s*_(*U*), where *w* should satisfy the condition: *w* = |*W*∗| = |*S*_*s*_(*U*)| < |*U*|.

Based on the above analysis, it can be concluded that *W*∗ must satisfy *W*∗ = sign(*U*) ⋅ min(*w*, |*U*|). Therefore, the optimal solution for *W* can be expressed as

(Equation 10) $$W^{*}\left(b\right)=sign\left(U\right)\cdot \min\left(M|b|,|U|\right)$$

Equation 6 shows that to find *b* in the optimization problem, it is only necessary to minimize the following objective:

(Equation 11) $$\begin{array}{cc} F\left(b\right) & =\frac{1}{2}\|r-b{\|}_{2}^{2}+\frac{1}{2}\|U-W\left(b\right){\|}_{F}^{2}+\alpha \lambda \|b{\|}_{1}+\left(1-\alpha \right)\lambda \|b{\|}_{2}\\ & =\frac{1}{2}\|r-b{\|}_{2}^{2}+\frac{1}{2}\|U-W\left(b\right){\|}_{F}^{2}+\lambda_{1}\|b{\|}_{1}+\lambda_{2}\|b{\|}_{2} \end{array}$$

At this point, let *w* = *M*|*b*|, and sort *U*_*i*_:

(Equation 12) $$\left|U_{i\left(1\right)}|\geq |U_{i\left(2\right)}|\geq \cdots \geq |U_{i\left(k\right)}\right|$$

Calculate the corresponding position when $w_{i}=M|b_{i}|\in \left(\left[\left|U_{i\left(s_{i}+1\right)}|,|U_{i\left(s_{i}\right)}\right|\right)\right)$, denoted as *m*_*i*_, then Equation 10 can also be written in the following form:

(Equation 13) $$W_{ij}=\left\{\begin{array}{cc} U_{ij}, & j>m_{i}\\ w_{i}\cdot sign\left(U_{ij}\right), & j\leq m_{i} \end{array}\right)$$

Combining the subgradient condition with basic algebra, taking the derivative of Equation 11 shows that if $\|S\left(\lambda -M\sum_{j=1}^{m}|U_{\left(j\right)}|,v\right){\|}_{2}\leq \lambda^{2}$, then *b*∗ = 0. Otherwise, *b*∗ should satisfy:

(Equation 14) $$\begin{array}{cc} \left(1+M^{2}m+\frac{\lambda_{2}}{\|b{\|}_{2}}\right)b & =v+\left(M\overset{s}{\underset{j=1}{\sum }}|U_{\left(j\right)}|-\lambda_{1}\right)sign\left(b\right)\\ & =S\left(\lambda_{1}-M\overset{m}{\underset{j=1}{\sum }}|U_{\left(j\right)}|,r\right) \end{array}$$

Let $\hat{S}$ in Equation 14 be $\hat{S}=S\left(\lambda_{1}-M\sum_{j=1}^{m}|U_{\left(j\right)}|,r\right)$, and then take the norm of both sides of the equation to obtain:

(Equation 15) $$\|b{\|}_{2}=\frac{{\left(\|\hat{S}{\|}_{2}-\lambda_{2}\right)}_{+}}{1+M_{2}m}$$

Substituting it into Equation 14, we can see that the solution of 11 with the generalized gradient step:

(Equation 16) $$b^{*}=\frac{\hat{S}{\left(\|\hat{S}{\|}_{2}-\lambda_{2}\right)}_{+}}{\left(1+M^{2}m\right)\|\hat{S}{\|}_{2}}$$

Thus, (16) and (10) give the solution to the objective function of the algorithm (5).

This method is implemented through Algorithm 1, and its specific steps are introduced below using data from one subject as an example. First, based on the loss function *L*(*θ*, *W*), the hierarchical sparse neural network performs an initialization training on the feedforward network. Next, the penalty term is initialized as *λ* = *λ*_0_ and the number of active features as *k* = *d*. Then, by computing pairwise correlations across the rs-fMRI time series **x**, the functional connectivity between ROIs is estimated. Based on these correlations and the original data, a static adjacency matrix is constructed to represent the overall brain network structure, which remains constant across time. Subsequently, this static graph is input into the spatio-temporal convolution module to generate node features **x**_**e**_ containing spatio-temporal information.

Algorithm 1 **Hierarchical Sparse Spatio-Temporal Graph Neural Network Model (GLNSTGNN)**

**Table:** 

**Input:** Training dataset $x\in R^{v\times T}$, feature groups $G=\left\{G_{1},\ldots ,G_{K}\right\}$, where $G_{i}$ contains the indices of features grouped together, the number of features in $G_{i}$ is *k*_*i*_, training set labels *y*, number of iterations *E*, hierarchical multiplier *M*, update multiplier *ϵ* for *λ*, learning rate *η*
1: Initialize and train a feedforward neural network based on the loss function *L*(*θ*, *W*).
2: Initialize the penalty parameter *λ* = *λ*_0_ and the number of features *k* = *d*.
3: Construct a static FC adjacency matrix using correlation coefficients computed from the full time series, and keep it fixed for all subsequent temporal modeling.
4: **while***k* > 0 **do**
5: The data *x* is input into the spatiotemporal convolution module for processing to obtain the node feature *x*_*e*_.
6: Update *λ* ← (1 + *ϵ*)*λ*
7: **for***e* ∈ {1, …, *E*} **do**
8: Use backpropagation to compute the gradients of the loss function with respect to (*θ*, *W*).
9: Update *θ* ←*θ* − *η*▽_*θ*_*L and W* ← *W* − *η*▽_*W*_*L*
10: **for**g∈G**do**
11: $\left(\theta_{\left(g\right)},{W_{\left(g\right)}}^{\left(1\right)}\right)\leftarrow GroupLassoNet-Prox\left(\theta_{\left(g\right)},{W_{\left(g\right)}}^{\left(1\right)},\eta \lambda ,\alpha ,M\right)$
12: **endfor**
13: **endfor**
14: Update *k* to the number of non-zero parameters in *θ*.
15: **endwhile**

Subsequently, the node features **x**_**e**_ are input into the hierarchical sparse neural network. In the subsequent iterative process, the penalty term *λ* will be gradually updated, and the weights and biases in the network will be optimized. In each iteration cycle, the algorithm calculates the gradients of the loss function with respect to the parameters *θ* and *W* through backpropagation, and updates the parameters using gradient descent. Meanwhile, in the feature group loop, for each feature group *G*, the relevant parameters are adjusted by invoking the GroupLassoNet-Prox optimization algorithm. Finally, based on the latest parameter *θ*, the number of non-zero coefficients *k* is recalculated. This process is repeated until the feature number *k* reaches zero or a preset value, and the algorithm terminates.

In line 6 of the algorithm, a warm-start strategy is employed to update *λ*. Initially, a small *λ* is set to allow the neural network to prioritize exploring paths to local optimal solutions. As training progresses, *λ* is gradually increased to promote feature sparsity. Additionally, the exponential growth of the sparse regularization term accelerates the algorithm’s convergence. The optimization process in line 11 is carried out using the GroupLassoNet-Prox method; detailed steps can be found in Algorithm 2.

Algorithm 2 **GroupLassoNet-Prox Optimization Algorithm**

**Table:** 

**procedure** GroupLassoNet-Prox$\left(b\in R^{k},W\in R^{k*K},\lambda ,\alpha ,M\right)$
**Notion:**
1: **for***j* ∈ 1, …, *k***do**
2: Sort $W_{j}^{\left(1\right)}\in R^{K}$ such that $\left|W_{j,\left(1\right)}^{\left(1\right)}|\geq \ldots \geq |W_{j,\left(K\right)}^{\left(1\right)}\right|$
3: **for***m* ∈ 0, …, *K***do**
4: $a_{m}=\alpha \lambda -M\sum_{i=1}^{m}|W_{j\left(i\right)}|$
5: ${\hat{S}}_{m}=S\left(a_{m},v\right)$
6: $w_{j,m}\leftarrow M*|\frac{{\hat{S}}_{m}{\left(\|{\hat{S}}_{m}{\|}_{2}-\left(1-\alpha \right)\lambda \right)}_{+}}{\left(1+M^{2}m\right)\|{\hat{S}}_{m}{\|}_{2}}|$
7: **endfor**
8: Find the first *m* ∈ {0, …, *K*} that satisfies $\left|W_{j,\left(m\right)}^{\left(1\right)}|\geq w_{j,w}\geq |W_{j,\left(m+1\right)}^{\left(1\right)}\right|$, denoted as ${\tilde{m}}_{j}$
9: **endfor**
10: $\tilde{m}=\left\{{\tilde{m}}_{1},{\tilde{m}}_{2},\ldots ,{\tilde{m}}_{k}\right\}$
11 $\hat{S}=\left\{{\hat{S}}_{1,{\tilde{m}}_{1}},{\hat{S}}_{2,{\tilde{m}}_{2}},\ldots ,{\hat{S}}_{k,{\tilde{m}}_{k}}\right\}$
12: **if**$\|\hat{S}{\|}_{2}\leq \left(1-\alpha \right)\lambda$**then**
13: *b*∗ ← 0
14: *W*^(1)^∗ ← 0
15: **else**
16: Update $b^{*}\leftarrow \frac{\hat{S}{\left(\|\hat{S}{\|}_{2}-\lambda_{2}\right)}_{+}}{\left(1+M^{2}\tilde{m}\right)\|\hat{S}{\|}_{2}}$
17: Update $W^{\left(1\right)*}\leftarrow sign\left(W_{j}^{\left(1\right)}\right)*min\left(w_{\tilde{m}},|W_{j}^{\left(1\right)}|\right)$
18: **endif**

#### Dataset preparation

The data we used were obtained from publicly available, standardized preprocessing pipelines provided by the Preprocessed Connectomes Project (PCP) as shown in key resources table.

**ABIDE Dataset:** The ABIDE dataset contains structural and rs-fMRI data collected from multiple international research centers, along with detailed phenotypic information for each subject, including age, sex, and diagnostic status, as summarized in the table below. After data quality control (removing subjects with excessive movement), the rs-fMRI data of 1,009 subjects were finally included, among which 516 were diagnosed with Autism Spectrum Disorder (ASD) and 493 were Typically Developing Controls (TC). For functional connectivity analysis, the Craddock 200 cortical network template (CC200) was used, which divides the entire brain into 200 nodal regions.^53^ After standard preprocessing steps, including temporal drift removal, spatial normalization, and Gaussian smoothing, the extracted time-series data ultimately contained 200 nodes, with the time series of each node having a length of 100 time points.

**Table:** Subjects from the ABIDE sample and their partial demographic information

ASD						TC			
Site	Age Avg	ADOS	Sex	Handedness	Verbal IQ	Age Avg	Sex	Handedness	Verbal IQ
CALTECH	27.4	13.1	M 15, F 4	R 14, A 5	107.7	28.0	M 14, F 4	R 14, L 1, A 3	114.4
CMU	26.4	13.1	M 11, F 3	R 12, L 1, A 1	111.2	26.8	M 10, F 3	R 12, A 1	112.8
KKI	10.0	12.5	M 16, F 4	R 16, L 1, A 3	∖	10.0	M 20, F 8	R 23, L 2, A 3	∖
LEUVEN	17.8	∖	M 26, F 3	R 26, L 3	99.45	18.2	M 29, F 5	R 29, L 4, A 1	116.1
MAX MUN	26.1	9.5	M 21, F 3	R 22, L 2	∖	24.6	M 27, F 1	R 28	∖
NYU	14.7	11.4	M 65, F 10	R 58, L 16, A 1	105.8	15.7	M 74, F 26	R 98, L 2	113.1
OLIN	16.5	14.1	M 16, F 3	R 16, L 3	∖	16.7	M 13, F 2	R 13, L 2	∖
PITT	19.0	12.4	M 25, F 4	R 25, L 3, A 1	107.3	18.9	M 23, F 4	R 26, L 1	107.7
SBL	35.0	9.2	M 15, F 0	R 11, L 4	110.4	33.7	M 15, F 0	R 13, L 2	∖
SDSU	14.7	11.2	M 13, F 1	R 13, L 1	110.1	14.2	M 16, F 6	R 19, L 3	106.7
STANFORD	10.0	11.7	M 15, F 4	R 14, L 4, A 1	109.2	10.0	M 16, F 4	R 18, A 2	111.2
TRINITY	16.8	10.8	M 22, F 0	R 22	108.5	17.1	M 25, F 0	R 25	109.6
UCLA	13.0	10.9	M 48, F 6	R 48, L 6	97.4	13.0	M 38, F 6	R 40, L 4	107.9
UM	13.2	∖	M 57, F 9	R 58, L 7, A 1	111.4	14.8	M 56, F 18	R 64, L 10	113.6
USM	23.5	13.0	M 46, F 0	R 39, L 7	96.0	21.3	M 25, F 0	R 23, L 2	112.9
YALE	12.7	11.0	M 20, F 8	R 22, L 6	96.5	12.7	M 20, F 8	R 24, L 4	106.8

Note: F/M: Female/Male, L/R/A: Left/Right/Ambidextrous, ∖: Unavailable, ADOS: Autism Diagnostic Observation Schedule score, VIQ: Verbal Intelligence Quotient score.

**ADHD Dataset:** The ADHD dataset is a multi-center joint data resource, containing rs-fMRI scans, structural MRI scans, and multiple phenotypic data from participants of different age groups. Participants in this dataset include healthy control individuals and patients diagnosed with Attention-Deficit/Hyperactivity Disorder (ADHD). After strict data quality screening (excluding participants with poor data quality), rs-fMRI data of 947 participants were finally selected, among which 362 were ADHD patients and the remaining were healthy control groups. Consistent with the ABIDE dataset, this dataset also uses the Craddock 200-node brain atlas for brain functional connectivity analysis. The preprocessed data includes 200 nodes, and each node is composed of time-series data of the first 120 time points.

The pipelines of ABIDE and ADHD include standardized procedures for motion correction, nuisance regression, and temporal filtering to ensure consistency and reproducibility. The preprocessing details is as following:

• Motion regression: Six rigid-body motion parameters and their derivatives were regressed out.
• WM/CSF/global-signal regression: White-matter, cerebrospinal-fluid, and global signals were removed following the default PCP implementation.
• Band-pass filtering: Temporal filtering was applied within the 0.01–0.1 Hz range to retain resting-state relevant fluctuations.
• Scrubbing: Frames exceeding the motion threshold (framewise displacement > 0.5 mm) were removed according to PCP’s default configuration.

We acknowledge that the ABIDE and ADHD datasets differ in their original scan lengths due to variations in acquisition protocols across contributing sites. To ensure comparability, we standardized the sequence length across all subjects. Specifically, we removed the initial unstable period of the rs-fMRI signal (first 5–8 seconds) during preprocessing.

**MNIST Dataset** (Modified National Institute of Standards and Technology) is a classic benchmark dataset widely used in handwritten digit recognition tasks.^54^ The dataset contains 70,000 grayscale images of handwritten digits, with each image having a resolution of 28× 28 pixels. The images depict handwritten forms of digits from 0 to 9. The dataset is divided into two parts: 60,000 images for training and 10,000 images for testing, ensuring the balance between training and testing.As shown in Figure S3, several example images from the MNIST dataset are displayed, all of which are black-and-white handwritten digits. The digits 0 to 9 in the figure are samples from different people’s handwriting, with certain deformations and noise, which poses challenges to the generalization ability of models.

### Quantification and statistical analysis

#### Sparse selection evaluation

To visually demonstrate the effectiveness of the GroupLassoNet method in sparse representation, this experiment constructs a new training sample set ***X*** based on the MNIST dataset. The MNIST dataset is a widely used benchmark dataset that contains handwritten digit images from 0 to 9. For this experiment, 1,000 images were first randomly selected from the MNIST dataset, covering 10 classes (digits 0 to 9). Each class was represented by 100 images to ensure class balance. All selected images were then converted into vector form and assembled into the training samples ***x***, specifically represented as:

$$x=\left[x_{0,1},\ldots ,x_{0,100},x_{1,1},\ldots ,x_{1,100},\ldots ,x_{9,1},\ldots ,x_{9,100}\right]$$

In this process, each image is regarded as an independent feature in the feature selection process. However, unlike the traditional approach of treating each pixel of an image as a separate feature, the image set ***x*** here focuses more on treating the image itself as a feature set for processing. It should be noted that this processing method is different from the traditional image feature extraction method, in which each pixel of each image is usually regarded as a feature dimension. In the experiment, an image from the category ’0’ was also randomly selected as the target *y*, which represents the label of the target class. Then, based on the feature set ***x*** selected from the MNIST dataset, multiple sparse representation techniques were adopted to generate sparse representations for the target *y*, and the distribution characteristics of the selected features and their relationships with the target *y* were analyzed.

In Figure S4, the horizontal axis of each subgraph represents 1000 image data, which are sorted according to the category numbers. For example, the interval 0-100 represents images of category ’0’, 100-200 represents images of category ’1’, and so on. The vertical axis represents the weight value of each feature. The weight values of all methods have been standardized before comparison to clearly demonstrate the effects of each method.

It can be observed that each sparse representation method exhibits distinct feature selection patterns. Both traditional sparse methods and the GroupLassoNet method proposed in this study show higher weights in the feature groups of the same category as the target y, especially in the ’0’ category. This indicates that these methods can effectively identify and retain features related to the target category. Notably, as a variant of group sparsity methods, GroupLassoNet can achieve both intra-group and inter-group sparsity simultaneously. Specifically, features in certain groups of the method completely vanish (such as Group 1, Group 4, Group 7, and Group 9), while only part of the features in other groups are retained. In contrast, the LassoNet and Lasso methods fail to exhibit obvious group sparsity characteristics. In these methods, features of each category retain a certain proportion of characteristics, suggesting that they cannot effectively achieve group sparsity.

The Group Lasso method only exhibits sparsity between groups, that is, it can selectively retain features of certain groups, but all features within the selected groups are retained, so it lacks the ability for more refined feature screening. In contrast, the Sparse Group Lasso method shows effects similar to the GroupLassoNet method, demonstrating better group sparsity in the selection of retained features, but its grouping effect is still less refined than that of the GroupLassoNet method.

These results indicate that the GroupLassoNet method, during the feature selection process of sparse representation, can not only automatically filter out features related to the target category but also achieve more precise feature extraction through effective intra-group and inter-group sparsity. The advantage of this method lies in its ability to achieve more efficient and interpretable feature selection in high-dimensional feature spaces, which holds important application value for image classification and other machine learning tasks.

#### Evaluation of sparse self-expression classification performance

To thoroughly evaluate the effectiveness of GroupLassoNet in sparse self-expression-based classification tasks, this experimental study examines the algorithm’s performance in identifying basis samples that are strongly correlated with the target sample and further assesses its confidence prediction capabilities. In image data classification tasks, samples within the same category typically share similar structural features, which results in strong correlations among these samples. Based on this premise, it is hypothesized that GroupLassoNet can effectively identify samples with high relevance to the target sample through sparse self-expression techniques and provide corresponding confidence scores. To achieve this goal, the confidence score is calculated using the formula:

(Equation 17) $$conf_{i}=\frac{\underset{j\in G_{i}}{\sum }w_{j}}{\overset{n}{\underset{j=1}{\sum }}w_{j}}$$

In Equation 17, *conf*_*i*_ represents the confidence of the *i*-th sample, *w*_*j*_ is the weight value of the feature, *G*_*i*_ indicates the feature group associated with the target sample, and *n* is the number of all features.

To compare the effects of different methods, the experiment calculated the confidence of each group of features in the experiment shown in Figure S4. The calculation method of confidence is to sum the weights of each group of features and divide by the sum of the weights of all features, as shown in the above formula. According to the results displayed in Figure S5, it can be seen that no matter which method is used, it can achieve a high confidence in its corresponding category. However, when conducting a comparative analysis, it is found that GroupLassoNet has achieved the highest confidence in fitting the corresponding category of the target sample. Compared with all other methods, this result has significant advantages. This indicates that GroupLassoNet may be the most reliable and accurate method for completing such tasks, further confirming its advantages in using group sparsity. Specifically, the high confidence obtained by GroupLassoNet in the corresponding category indicates that it can effectively identify the important features within the group, which is unattainable by other methods.

This advantage of GroupLassoNet not only enhances the model’s interpretability but also further improves its generalization ability in handling real-world problems. This feature has potential application values in multiple fields such as bioinformatics, financial analysis, and social sciences. By adopting group sparsity, GroupLassoNet can provide more accurate and interpretable results in various tasks.

Furthermore, considering the advantages of GroupLassoNet in confidence calculation, this study decided to use the softmax function to evaluate confidence and perform classification based on it. Traditional classification models typically directly use class labels as *y* and apply sparsity methods to simplify the model or screen relevant features. However, in this experiment, sparse self-expression learning is directly performed on independent test samples, the weights of their sparse representations are calculated, the training samples most strongly associated with the test samples are automatically selected, and classification is performed based on the results of the sparse representations. In other words, the sample *y* is classified into the category with the highest confidence score. A series of experiments were conducted based on this self-expression method.

First, 100 images from 10 categories were randomly selected from the MNIST dataset, totaling 1,000 images as the sample set *X*. When using the group sparsity method, the 1,000 images were divided into 10 categories, with 100 images per category as one group. Meanwhile, 300 images were selected as the target *y*. Then, the sparse representation method was applied to calculate the confidence score of each target *y* on the sample set *X*, and it was classified into the category with the highest confidence. Finally, the classification accuracy was calculated based on these classification results.

To further evaluate the performance of the GroupLassoNet method, the accuracy of the classification task was calculated. The calculation process of accuracy is as follows: for each test sample, it is classified into the category with the highest confidence score. Then, the ratio of the number of correctly classified samples to the total number of samples (*n*/*N*) is calculated as the classification accuracy. This metric can objectively evaluate the performance of the proposed method in classification tasks. A higher accuracy indicates that GroupLassoNet can effectively classify samples into the correct categories, thus demonstrating its practicality and accuracy in classification tasks.

In addition, among all correctly classified samples, the average confidence was also calculated, with the formula as follows:

(Equation 18) $$conf_{\mathrm{avg}}=\frac{1}{n}\underset{i\in n}{\sum }conf_{i}$$

Here, *n* is the number of correctly classified samples, and *conf*_*i*_ is the confidence level of the *i*-th correctly classified sample.

Figure S6 shows a comparative analysis of accuracy and average confidence in classification tasks based on sparse self-expression, covering a variety of linear and nonlinear methods. Through the analysis of the data in the figure, it can be found that GroupLassoNet performs excellently in both key indicators of accuracy and average confidence, and is superior to other methods.

These experimental results reveal the excellent performance of GroupLassoNet in sparse self-expression classification tasks. Compared with other linear and nonlinear methods, GroupLassoNet integrates the characteristics of residual deep learning networks, enabling it to more effectively identify the interrelationships between samples, thereby improving the accuracy and confidence of classification tasks. This advantage endows GroupLassoNet with extensive application potential in multiple fields, especially when dealing with high-dimensional sparse data.
