# Supplementary material for: Hierarchical sparse spatiotemporal graph neural network for brain graph classification
Source: iScience. 2026 Jun 4;29(6):116173. doi: 10.1016/j.isci.2026.116173 (PMC13266135; doi:10.1016/j.isci.2026.116173)
Supplement: Document S1. Figures S1–S6 [file mmc1.pdf]

iScience, Volume *29*

Supplemental information

Hierarchical sparse spatiotemporal graph neural network for brain graph classification

Jiaqi Cui, Yuxin Li, Xiran Qu, and Yupei Zhang

Supplemental Figures and Legends

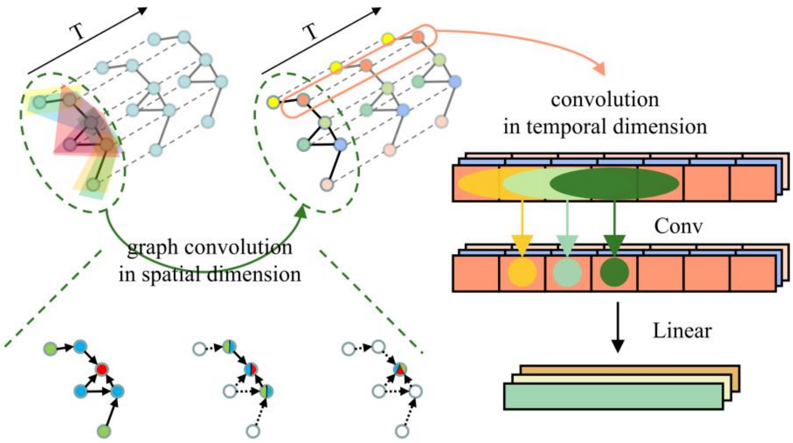

Figure S1. Spatio-Temporal Convolution Module

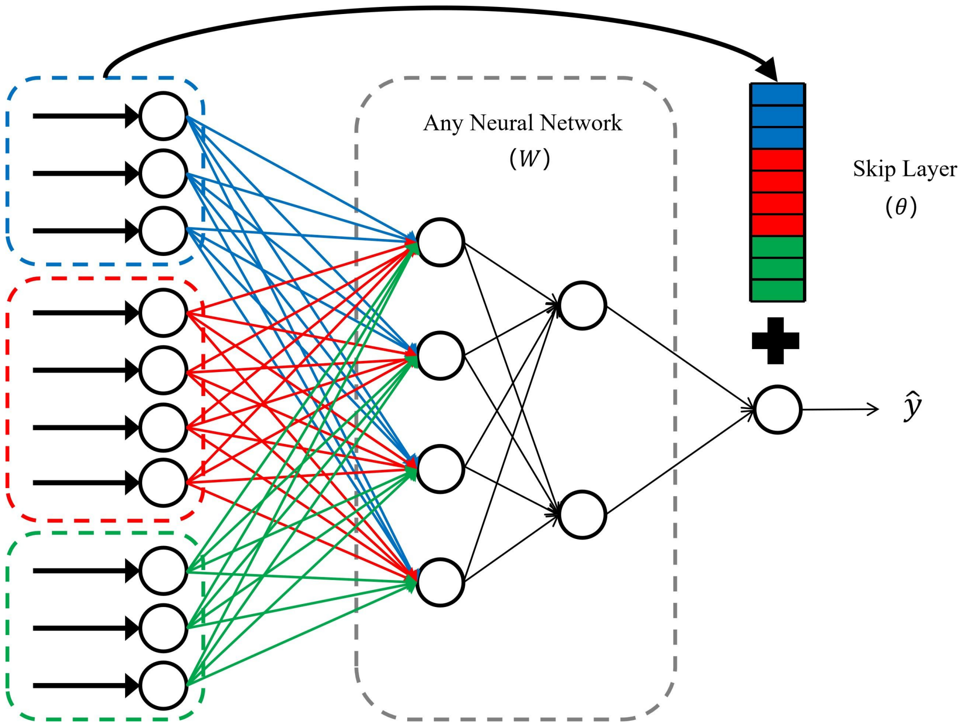

Figure S2. GroupLassoNet Model Framework

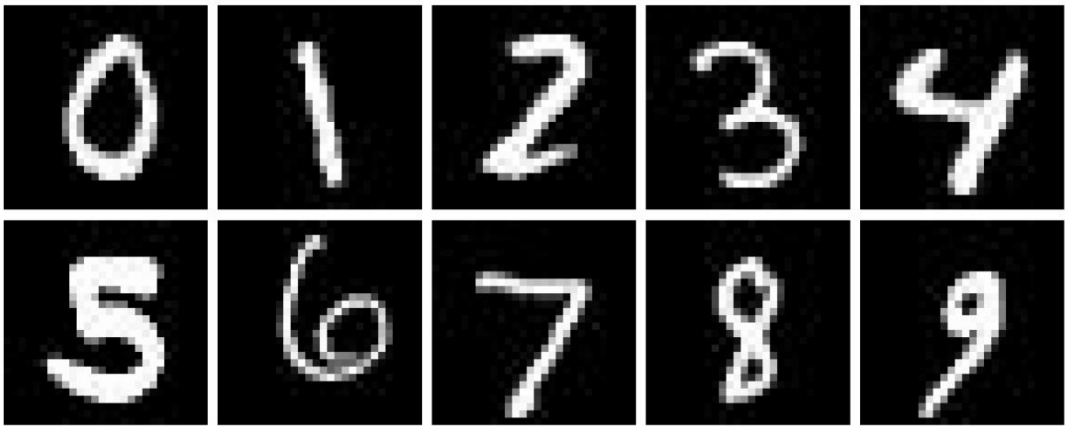

Figure S3. Example Samples of the MNIST Dataset

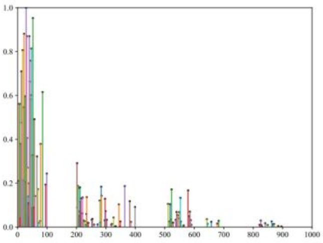

(a) GroupLassoNet

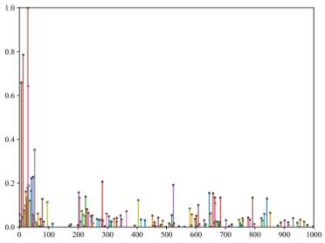

(b) LassoNet

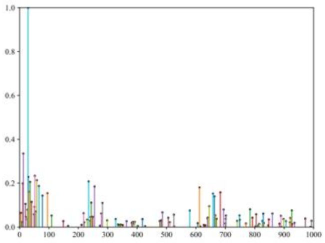

(c) Lasso

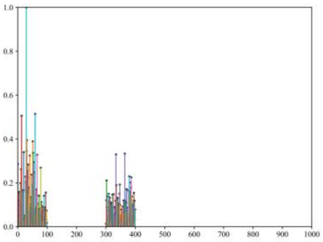

(d) Group Lasso

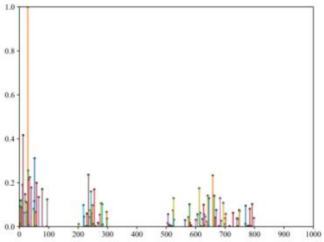

(e) Sparse Group Lasso

Figure S4. The Feature Selection Results on MNIST

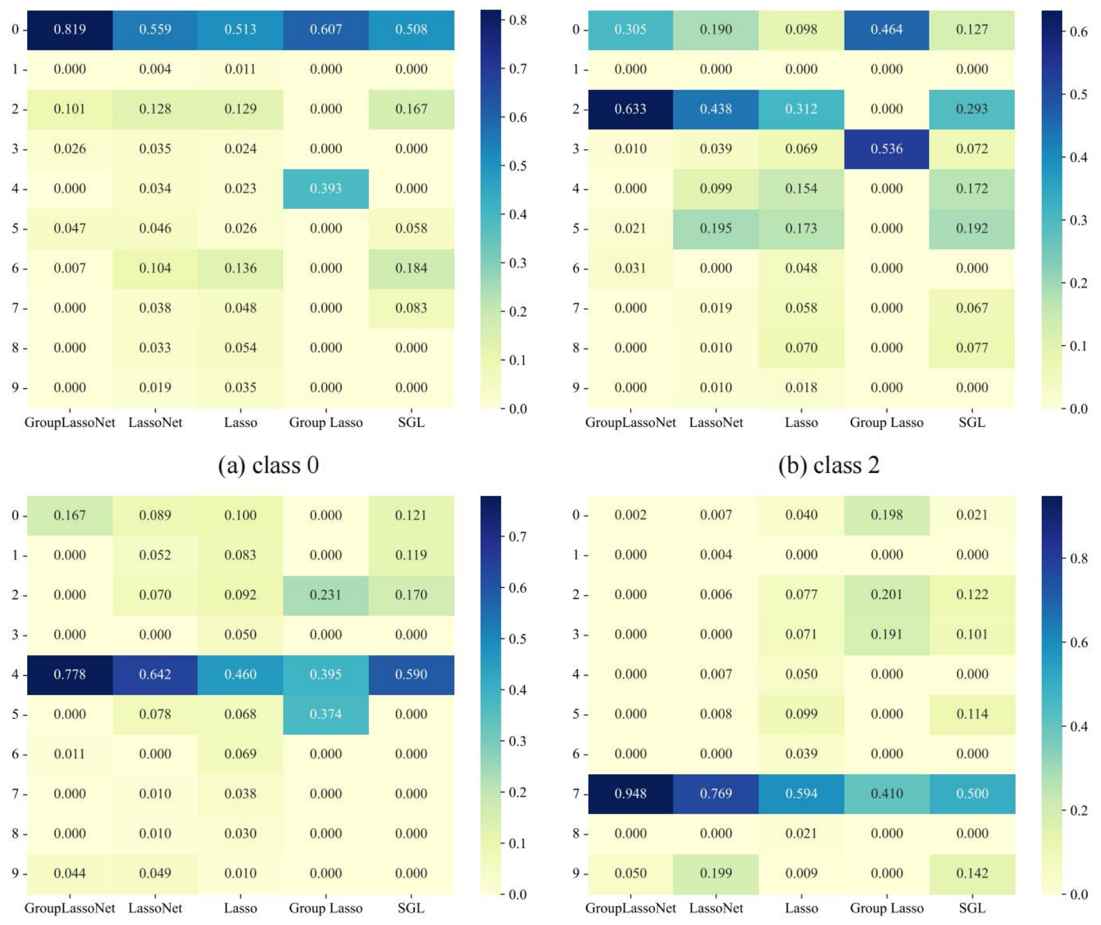

(a) class 0

(b) class 2

(c) class 4

(d) class 7

Figure S5. The Confidence When Fitting Different Categories of the MNIST Dataset

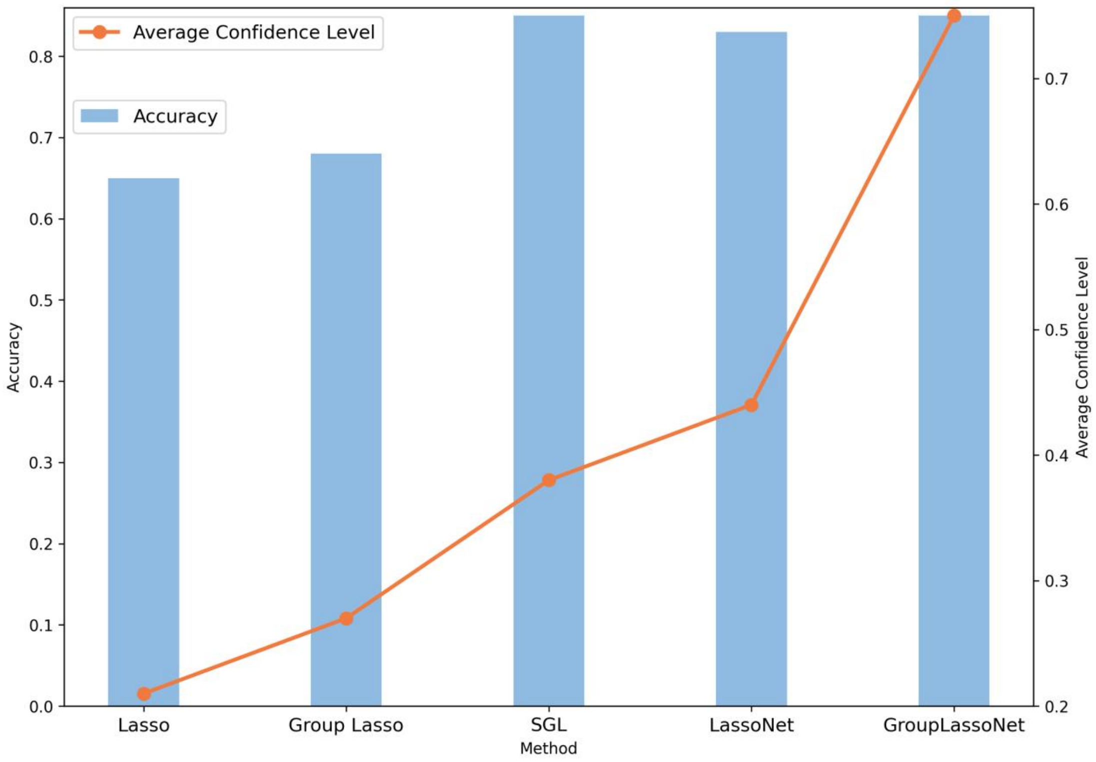

Figure S6. Classification Accuracy and Its Average Confidence Level Based on Sparse Representation
